# The managers' perspectives on service providing in women's harm reduction centers during the COVID-19 pandemic: mixed method study

**DOI:** 10.1186/s12954-024-01049-z

**Published:** 2024-07-12

**Authors:** Azam Rahmani, Maryam Janatolmakan, Elham Rezaei, Malihe Tabarrai

**Affiliations:** 1 Nursing and Midwifery Care Research Center, School of Nursing and Midwifery, Tehran University of Medical Sciences, Tehran, Iran; 2 Social Development and Health Promotion Research Center, Health Institute, Kermanshah University of Medical Sciences, Kermanshah, Iran; 3 Nursing and Midwifery Care Center, Tehran University of Medical Sciences, Tehran, Iran; 4 Midwifery Department, School of Nursing and Midwifery, Tabriz University of Medical Sciences, Tabriz, Iran; 5 Department of Persian Medicine, School of Persian Medicine, Tehran University of Medical Sciences, Tehran, Iran

**Keywords:** Harm reduction, COVID-19, Mixed method, High-risk women, Managers, HIV

## Abstract

**Background:**

The COVID-19 pandemic posed significant challenges for managers overseeing women's harm reduction centers. This study seeks to capture managers' perspectives on the service providing in women's harm reduction centers during the COVID-19 pandemic.

**Methods:**

This convergent mixed-method study conducted in three provinces of Iran: Tehran, Khuzestan, and Kermanshah. The study was carried out between January and May 2023. In the quantitative part, the researchers utilized reports from 10 center managers. A researcher-designed questionnaire was employed to collect data on a wide range of services and referrals provided by the centers. The qualitative part of the research involved conventional content analysis and included 12 individual interviews. Two directors from the Ministry of Health and ten managers of women’s harm reduction centers participated in the interviews. During the interpretation phase, the researchers compared the quantitative and qualitative findings to obtain a comprehensive understanding of the topic.

**Results:**

During the quantitative stage of the study, it was observed that all the managers were women, with an average age of 40.7 ± 7 years. More than half of the managers had obtained a postgraduate education (n = 6, 60%), and a majority of them were married (n = 7, 70%). Additionally, 40% of the managers (n = 4) were working as contractors. During the non-COVID-19 period, there were higher coverage by centers and residents, more referrals of non-injecting drug users and sex workers, and a higher number of group counseling sessions in all three provinces compared to the COVID-19 period. The qualitative analysis revealed two primary themes: "challenges" and "capabilities."

**Conclusions:**

During the COVID-19 pandemic, providing some services and client referrals decreased in the centers, and center managers faced increased challenges. Many of these challenges were in the communication, executive, management, structure, education, financial, civilization, facilities, and socio-cultural sectors. Managers used their skills to manage and control these challenges. It is important to focus on these challenges and managerial capabilities to effectively handle future crises.

## Introduction

The COVID-19 pandemic has had a global impact on healthcare systems, including harm reduction centers [1]. During the COVID-19 pandemic, women's harm reduction centers face added challenges due to their vulnerable clients, who often engage in high-risk behaviors, and also the related stigma existing for both health providers and health givers [2, 3]. Community centers play a vital role in providing essential services such as HIV prevention, distribution of harm reduction supplies (e.g., syringes, needles, and condoms), and counseling. Their ability to adjust to the COVID-19 pandemic and continue delivering these crucial services is of utmost importance [4, 5]. Many managers worldwide have adopted innovative strategies to mitigate the impact of the pandemic on their operations. These measures include deploying mobile service teams, offering virtual services, empowering women through interventions, providing home-based services, utilizing digital technologies for training, and implementing automated dispensers for harm reduction equipment distribution [3, 6–10]. Despite the efforts of managers to handle the COVID-19 pandemic, they encountered several challenges. These included financial constraints, disruptions in service processes, staffing shortages, reduced working hours, center closures, lack of protective equipment, social isolation of patients, limitations in follow-up, insufficient food and shelter, lack of diagnostic kits, and unequal distribution of vaccines. These challenges have hindered their ability to provide adequate care and support to those in need during this time [2, 3, 6, 11, 12]. Despite the challenges, harm reduction centers have managed to continue providing primary services to clients without significant interruptions [13, 14]. However, some studies have reported decreased service provision and referrals due to COVID-19 [15–18]. In Iran, a qualitative study explored the perspectives of policymakers, service providers, researchers, and clients on the impact of the pandemic on HIV/AIDS-related services. The findings indicated severe initial effects that gradually diminished over time, although diagnostic and preventive services were more severely affected than others [12]. In 2022, a qualitative study was conducted in Iran to examine the efforts of non-governmental organizations in serving vulnerable individuals during the pandemic [19]. Several qualitative studies in Iran have focused on identifying the activities and challenges faced by non-governmental organizations in response to the pandemic [20]. These studies primarily involved representatives of these organizations. Furthermore, a framework was developed based on the achievements and experiences of these organizations to implement effective measures in other communities [21]. Although several qualitative and descriptive studies have been conducted in Iran [12, 19–21] from the perspectives of managers and employees of non-governmental organizations, no multicenter and mixed method study has yet investigated the viewpoints of women's harm reduction center managers. Additionally, no study has examined the services provided by these centers before and after the COVID-19 pandemic. Therefore, the current research was designed to investigate managers' perspectives on providing services in women's harm reduction centers during the COVID-19 pandemic.

## Materials and methods

### Study design and settings

This study employed a convergent mixed methods approach, integrating quantitative and qualitative methods to enhance the credibility and comprehensiveness of the findings [22]. The research aimed to investigate managers' perspectives on providing services in women's harm reduction centers during the COVID-19 pandemic. We combined two research methods for a comprehensive approach. The research was conducted in two parts: a quantitative part and a qualitative part, conducted separately. The sample for the study consisted of senior organizational managers and center managers located in Tehran, Khuzestan, and Kermanshah provinces. Due to the higher number of clients and increased vulnerability in these provinces, these three provinces were intentionally chosen for the study over others in the country. The selection of managers was based on their performance and service history both before and after the onset of the COVID-19 pandemic. In addition, the centers were selected from specific provinces due to the high number of affected women visiting the centers. These centers provided services such as distribution of needles, syringes, needle tips, and condoms, as well as regular screenings and vaccinations for women engaging in high-risk behaviors. Social worker, psychiatrists, psychoanalysts, nutrition teams, and administrative staff work at these centers. Their education levels range from diplomas to university degrees. These centers cater to women exhibiting high-risk behaviors.

### Participants and data collection

This research was carried out from January to May 2023. The inclusion criteria for participants were their willingness to take part in the study and their experience in managing centers before and during the COVID-19 pandemic. Regarding the quantitative part of the study, we invited the managers responsible for ten women's harm reduction centers to participate. Quantitative data were collected directly from questionnaires completed by the researcher and center managers, since these data has a sensitive nature, the researcher were not allowed to check the records lonely. For the qualitative section of the study, a purposive sampling method was employed to select participants for individual interviews. Specifically, two managers from the Ministry of Health, Treatment, and Medical Education in Tehran, as well as ten managers overseeing women's harm reduction centers across five centers in Tehran, three centers in Khuzestan, and two centers in Kermanshah were approached to participate in the qualitative component of the study. The decision to involve two senior managers from the Ministry of Health was based on their knowledge and experience toward the conditions at women's harm reduction centers during the COVID-19 pandemic. Their critical experiences and viewpoints helped us gain a better understanding of that period.

### Study tools

During the quantitative phase of the study, a questionnaire was utilized by the research team to assess changes in service delivery before and during the COVID-19 pandemic. Quantitative data was solely extracted from the written reports of the managers because we needed the overall statistics from two periods, COVID and non-COVID periods. The questionnaire consisted of two parts. The first part collected demographic information such as age, marital status, education level, and employment status. The second part included questions pertaining to the number of patient referrals, HIV prevention services, counseling services, and COVID-19-related services provided by the centers under the managers' supervision both during and prior to the pandemic. In the survey, we considered the COVID-19 pandemic period in Iran (2020 February -2023 July), and the non-pandemic period (before December 2019, and from August 2023 onwards). In the qualitative section of the research, semi-structured interviews were conducted to gather data. Some of the questions of the interviews include: (1) how would you describe the quality and quantity of services at women's harm reduction centers during and before the COVID-19 pandemic? (2) Did you have any changes in the management and structure of the centers during the COVID-19 pandemic? If so, have these changes led to challenges, problems, or achievements that you can discuss?

Exploratory questions were also asked based on the participants' responses to each main question to gain a deeper understanding of their experiences. Data collection continued until data saturation was achieved. A total of 12 interviews were conducted, and during the last interview, no new information or insights were obtained from the managers. The interviews were conducted by the first author of the study, an expert in conducting qualitative research and an associate professor at the university. The time of interviews were scheduled by phone calls. The interviews lasted 45–60 min and took place at a meeting room of the Ministry of Health and a private room of the centers.

### Data analysis

Quantitative and qualitative data were collected concurrently but analyzed separately. For quantitative analysis, the SPSS statistical software program, version 19, was employed. Descriptive statistics, such as mean (standard deviation) and frequency (percentage), were used to describe the quantitative variables.

The qualitative part of the study involved transcribing and analyzing each interview in a step-by-step manner. The analysis approach proposed by Granheim and Lundman [23], was employed, where the entire interview served as the most appropriate unit of analysis. Meaningful units, such as words, sentences, or paragraphs, were identified and classified as semantic units based on their meaning. Each semantic unit was then assigned a name reflecting its explicit or implicit meaning. Categories were compared to identify differences and similarities, which led to the emergence of final themes. The Max-QDA software was utilized for qualitative data analysis.

During the interpretation phase of the study, the quantitative and qualitative data were compared to gain a more comprehensive understanding of the research findings. This process involved examining both consistent and inconsistent data from both types of analysis. By considering the outcomes of both approaches, a more complete picture of the study was obtained.

### Trustworthiness

To ensure the reliability of the study, several measures were implemented. Firstly, the interviews were conducted by an experienced expert in the field to maintain their quality and accuracy. Secondly, the four participating managers were asked to provide feedback on the codes extracted from their interviews, which enhanced the validity and reliability of the findings. Additionally, external researchers with knowledge of qualitative content analysis independently reviewed and evaluated the codes and themes. This external validation process was a crucial step taken to confirm the accuracy and reliability of the study. To enhance the transferability of the findings, a maximum diversity sampling method was employed. This method involved selecting managers of different ages, educational backgrounds, work experiences, and various organizational positions. By adopting this approach, a broader representation of the perspectives of center managers was achieved, increasing the generalizability of the findings to similar contexts.

### Ethical considerations

Ethical considerations were carefully addressed in the study, adhering to the guidelines of the Ethics Committee of Tehran University of Medical Sciences (IR.TUMS.FNM.REC.1401.175). The participants were fully informed about the research objectives, provided their informed consent, and their privacy and confidentiality were protected. They were also informed of their right to withdraw from the study at any point.

## Results

The research findings are divided into two main sections: quantitative and qualitative. The quantitative section presents specific data, such as the average age of women's harm reduction center managers, which was 40.7 ± 6.7 years. All managers in the study were women. More than half of the managers (n = 6, 60%) held postgraduate degrees, and the majority of them (n = 7, 70%) were married. Four managers (40%) worked as contractors, as shown in Table 1.

**Table 1:** Sociodemographic characteristics of managers of women's harm reduction centers

Qualitative part of the study (n = 12)^+^		Quantitative part of the study (n = 10)
		Variables, N (%)^++^
Age (Mean (SD))^+++^	42.3 (7.5)	40.7 (6.7)
Education		
Bachelor	4(33.3)	4(40.0)
Master	6(50.0)	6(60.0)
PhD	2(16.7)	–
Marital status		
Single	3(25.0)	3(30.0)
Married	9(75.0)	7(70.0)
Gender		
Female	10(83.3)	9(90.0)
Male	2(16.7)	1(10.0)
Employment status		
Informal	10(83.3)	10(100.0)
Formal	2(16.7)	–
Organizational position		
Managers of centers	10(83.3)	10(100.0)
Ministry managers	2(16.7)	–

^+^In addition to ten managers of harm reduction centers, two managers with ministerial organizational positions were also interviewed; ^++^ Number (percentage); ^+++^ Mean (standard deviation)

The results indicate that before the COVID-19 pandemic, the number of referrals, injecting drug users, and individual consultations in Tehran and Kermanshah provinces was higher compared to during the pandemic. However, the opposite trend was observed in Khuzestan province. In all three provinces, the number of people residing in the centers, non-injection drug clients, sex worker clients, and group consultations was higher during the non-pandemic period compared to the pandemic period. In Tehran province, the number of HIV-positive clients, HIV diagnostic tests, midwife visits, and medical visits did not differ significantly between the pandemic and non-pandemic periods of COVID-19. However, in Khuzestan and Kermanshah provinces, there were variations in these figures across the two time frames. Regarding the delivery of condoms and needles and syringes, in Tehran province, the quantities during the non-pandemic period were higher compared to the pandemic period. However, in Khuzestan and Kermanshah provinces, the figures showed differences between the two considered time frames, as presented in Table 2.

**Table 2:** The annual frequency of patient referrals and the services provided in harm reduction centers before and during the COVID-19 pandemic based on the report of the center managers

Variables (n^+^)	Provinces		
	Tehran	Khuzestan	Kermanshah
Referrals (general)			
COVID-19^++^	1888	800	120
Non-COVID-19^++^	2138	639	236
Residents			
COVID-19	645	80	–
Non-COVID-19	740	86	20
Number of people covered			
COVID-19	805	647	120
Non-COVID-19	1021	687	236
Injection drug users			
COVID-19	–	9	–
Non-COVID-19	3	8	19
Non-injecting drug users			
COVID-19	42	260	–
Non-COVID-19	255	282	113
Sex workers			
COVID-19	157	308	120
Non-COVID-19	553	323	226
HIV^+^			
COVID-19	6	29	1
Non-COVID-19	6	6	2
HIV diagnostic tests			
COVID-19	1232	809	60
Non-COVID-19	1232	857	264
Condom package provide			
COVID-19	163,560	30,430	–
Non-COVID-19	174,111	29,990	6454
Needle head and syringe provide			
COVID-19	1000	7575	–
Non-COVID-19	7785	7575	3302
Psychological counseling (optional service)			
Individual			
COVID-19	1505	915	720
Non-COVID-19	1721	688	836
Group			
COVID-19	49	24	–
Non-COVID-19	97	48	3
Midwifery visit			
COVID-19	50	384	–
Non-COVID-19	50	363	–
Medical visit			
COVID-19	48	142	–
Non-COVID-19	48	95	–
Psychiatric visit (optional service)			
COVID-19	–	42	–
Non-COVID-19	–	19	–

^+^Number, ^++^In the survey, we considered the COVID-19 pandemic period in Iran (2020 February–2023 July), and the non-pandemic period (before December 2019, and from August 2023 onwards)

According to the results, COVID-19 vaccination services were available twice in Tehran and Khuzestan provinces, while these services were not provided in the centers of Kermanshah province. On the other hand, COVID-19 screening was conducted twice in Tehran province and once in Khuzestan and Kermanshah provinces, as shown in Table 3.

**Table 3:** The annual frequency of services related to COVID-19 in women's harm reduction centers based on the report of center managers

Variable	People with/suspected of COVID-19 (n)^+^	Training related to COVID-19 (n)^+^	Vaccination (f)^++^	COVID-19 test (f)^++^
Tehran	100	436	2	2
Khuzestan	21	569	2	1
Kermanshah	30	3	–	1

+Number; ^++^Frequency

In the qualitative section of the study, the researchers conducted interviews with managers of women's harm reduction centers in three provinces, along with two senior managers at the ministerial level Table 1. The analysis of the interviews in the qualitative section resulted in 168 codes, which were used to extract two themes, twelve categories, and thirty-two subcategories. The themes identified were "challenges," and "capabilities." (Table  4)

**Table 4:** Themes, categories and subcategories of managers' views on the effects of COVID-19 on the services provided by women’s HIV harm reduction centers

Themes	Categories	Subcategories
Challenges	Communications	Indirect international communication
		limited virtual networks
		Restrictions of organizational communication
	Executive challenges	Inflexible upstream programs
		Absence of executive prerequisites
		Inaccessibility to services
		Discouragement in creativity
	Management	Lack of freedom in management
		Responsibilities shift
	Structure	Lack of organization chart
		Uncodified duties
		Labor shortage
	Education	Lack of international training workshops
		Ineffective learning strategies
		Ignoring training needs
	Finance and credit	Poor financial management
		Inadequate budget allocation for public screening
	Civilization	Lack of legal and judicial support
		Job insecurity
	Facilities	Lack of adequate equipment for healthcare
		Accommodation problems
	Socio-cultural challenges	Public panic
		Negative perceptions of the center and of patients
		Failure to respect human dignity
Capabilities	Supervision	COVID-19 registry
		Staff vaccinations
		Clients screening
	Executive capabilities	Mobile service teams
		PPE* manufacturing
		Urgent patient referral service
	Tact and diplomacy	Attracting crowdfunding
		Attracting international aids

^*^Personal Protective Equipment

1. Challenges
  The COVID-19 pandemic has led to an increased challenges for managers in various areas. This study has categorized the challenges expressed by managers into nine categories: “communications", "executive challenges", "management", "structure", "education", "finance and credit", "civilization", “facilities”, and “socio-cultural challenges”. Each category will be discussed in detail.
  1-1 Communications
    Crises such as the COVID-19 pandemic pose significant threats to countries. Effectively managing such crises requires establishing positive and productive relationships with both upstream and downstream entities involved. Communication has been identified as a key challenge among managers, encompassing several subcategories: indirect international communication, limited virtual networks, restrictions of organizational communication.
    1-1-1 Indirect international communication
      In our country, the services provided by the United Nations (UN) and the World Health Organization (WHO) are managed through the Ministry of Health. However, some participants expressed concerns about this arrangement and emphasized the need for direct communication with these international organizations.
      "*A major issue during the COVID-19 pandemic was the lack of direct communication with international institutions. This resulted in problems and executive needs not being effectively addressed in the centers*." (Participant 1).
    2-1-1 Limited virtual networks.
      During the COVID-19 pandemic, virtual services became a popular solution to prevent public gatherings. However, in Iran, the absence of a suitable platform for establishing virtual communication posed challenges.
      "*Unfortunately, the limited bandwidth of the country's internet and the filtering of some media messages during the COVID-19 pandemic caused difficulties in communicating with our clients and personnel*." (Participant 11).
    3-1-1 Restricted of organizational communication.
      Harm reduction centers faced challenges in referring sick patients to specialized treatment centers during the COVID-19 pandemic due to the need for inter-organizational and inter-departmental coordination.
      "*Delivering patients from our centers to hospitals was difficult, especially when the patient had a wound or a problem unrelated to COVID-19*."
      *“Our centers face an issue where the individuals responsible for making plans are not fully aware of our capabilities and resources. Consequently, they sometimes create programs that cannot be fully implemented in our centers. It would be beneficial if the managers of harm reduction centers could contribute their expertise to the planning process.*" (Participant 1).
  2-1 Executive challenges
    Managers encounter various challenges and difficulties when planning and implementing crisis management strategies. In this study, managers have discussed the challenges they face in the executive field. These challenges include: inflexible upstream programs, absence of executive prerequisites, inaccessibility to services, and discouragement in creativity. The subcategories will be further explained in the following sections.
    1-2-1 Inflexible upstream programs
      Managers reported that the inflexible nature of the programs communicated by the Ministry of health to the centers leaded to a significant challenge for them.
      "*Some of the programs and regulations we receive from higher authorities cannot be practically implemented due to insufficient facilities and resources available to us. Therefore, these programs should be designed in a flexible manner to allow for adaptability and implementation to the best of our abilities*." (Participant 9).
    2-2-1 Absence of executive prerequisites
      Managers believe that achieving desired goals requires proper preparation and meeting certain requirements. The lack of these requirements creates challenges for them.
      "*During the COVID-19 screening process, it was recommended that everyone undergo PCR testing. However, implementing this protocol encountered obstacles due to various reasons such as a shortage of personnel, lack of financial resources, and insufficient availability of test kits. Therefore, successfully implementing this protocol depended on providing the necessary prerequisites to our centers.*" (Participant 2).
    3-2-1 Inaccessibility to services
      According to the managers' report, women's harm reduction centers faced difficulties in providing services during the COVID-19 pandemic.
      "*Due to the reduced availability of test kits during the COVID-19 pandemic, our centers experienced delays in providing HIV screening services to clients*." (Participant 12).
    4-2-1 Discouragement in creativity
      Crises can sometimes lead to positive outcomes for societies, such as the discovery of hidden talents and the emergence of creativity and innovative ideas. However, during the COVID-19 pandemic, one of the challenges faced by contributors was the lack of support from higher-level individuals in the organization for their innovative ideas.
      "*I suggested using mobile vans in different parts of the city instead of fixed DICs, which may take up more space and limit access for clients. Unfortunately, this idea did not receive widespread support*." (Participant 1).
  3-1 Management
    Managers play a crucial role in the society, but they face various challenges during crises that can hinder proper management. These challenges can be categorized into two main areas: " Lack of freedom in management" which refers to their inability to make independent decisions, and " responsibilities shift" which is related to not having specific and fixed roles and responsibilities. By addressing these challenges, organizations can create a better working environment for managers, leading to enhanced overall productivity.
    1-3-1 Lack of freedom in management
      Managers sometimes feel that their freedom of action is restricted in their role, but with proper and timely support, it is possible to foster creativity, self-fulfillment, self-control, and independent thinking and action.
      "*During the COVID-19 pandemic, our upper management provided us with protocols and instructions that were sometimes challenging to fully implement at our centers. In my opinion, they should have given us more freedom to determine how to implement these measures*." (Participant 1).
    2-3-1 Responsibilities shift
      According to managers, having multiple roles and responsibilities hinders their ability to make optimal decisions and exercise their executive power, resulting in subpar performance within organizations.
      "*During the COVID-19 pandemic, we experienced the busiest period in our working lives. In addition to our regular duties, we also had to adhere to strict protocols and guidelines related to COVID-19. Due to a lack of resources and facilities, we had to exert extra effort to coordinate and prepare the necessary requirements.*" (Participant 11).
  4-1 Structure
    During the discussion on the structural aspect, managers identified three subcategories: " lack of organizational chart," " uncodified duties," and " labor shortage."
    1-4-1 Lack of organizational chart
      The organizational chart serves as a valuable tool for management as it provides a clear overview of the organization's structure. It outlines the relationships between different job positions, bringing order to work processes. Recently, one manager expressed concern about the absence of a formal organizational chart for harm reduction centers.
      "*If our centers were included in the Ministry of Health's organizational chart, it would address many of our issues and provide us with greater formality. This would also enable us to secure funding for our organization and create additional positions for personnel*." (Participant 1).
    2-4-1 Uncodified duties
      According to the managers, problems arise when the scope of activities of supervised centers does not align with their organizational structure.
      "*Our challenge was screening and vaccinating clients, which diverted our attention from our primary services. This task should have been assigned to an external team*." (Participant 4).
    3-4-1 Labor shortage
      Managers of the centers encountered a shortage of manpower during the COVID-19 pandemic.
      "*The lack of personnel during COVID-19 made it difficult to deploy mobile service teams, resulting in a decrease in their operations within the city*." (Participant 7).
  5-1 Education
    Failure to identify the educational needs of community members and health staff is considered a significant challenge for sustainable development and achieving organizational goals. Managers have explained three subcategories in the educational domain: " lack of international training workshops,” “ineffective learning strategies," and " ignoring training needs."
    1-5-1 Lack of international training workshops
      Managers have expressed a lack of comprehensive support during the COVID-19 pandemic as a challenge. They received more assistance with equipment and supplies rather than training.
      "*The UN provided support in terms of equipment only. They should also offer educational support by providing training for personnel and managers at all levels. This would help my staff better understand the importance the UN places on addressing COVID-19*." (Participant 2).
    2-5-1 Ineffective learning strategies
      Managers faced challenges in using traditional training methods during the COVID-19 pandemic.
*"COVID-19 has altered educational priorities and communication methods. The use of traditional methods posed challenges, necessitating alternative approaches such as semi-centralized, decentralized, and virtual education tailored to specific communities." (Participant 1).*
    3-5-1 Ignoring training needs
      Lack of employee training needs assessment was a challenge faced by managers during the COVID-19 pandemic.
      "*During COVID-19, guidelines were frequently changing, causing confusion among us and our staff. We need to assess our educational needs to enhance knowledge and prevent confusion.*" (Participant 10).
  6-1 Finance and credit
    The managers have raised two Challenges in the field of finance and credit: " poor financial management " and " inadequate budget allocating for public screening."
    1-6-1 Poor financial management
      During the COVID-19 pandemic, managers cited poor financial management as a significant challenge.
      "*The budget for our centers is small, and managers struggle to handle it due to inflation. The allocation of the centers' budget within the organization needs to be reviewed.*" (Participant 2).
    2-6-1 Inadequate budget allocation for public screening
      The COVID-19 pandemic has placed a strain on countries' resources for screening, vaccination, and treatment. Harm reduction centers have faced challenges due to limited facilities. It is necessary to allocate a separate budget for crises like this. Here are some participants' views:
      "*The implementation of the COVID-19 screening directive for affected women was challenging due to resource constraints*." (Participant 11).
  7-1 Civilization
    Managers referred to the civil challenges during the pandemic with subcategories such as "lack of legal and judicial support,”, and "job insecurity."
    1-7-1 Lack of legal and judicial support
      Lack of legal and judicial support presents a challenge for harm reduction center managers dealing with high-risk clients.
      "*Due to resource shortages during the pandemic, we were unable to fully implement all protocols. Legal support was lacking when it was needed*." (Participant 5).
    2-7-1 Job insecurity
      According to some managers, job insecurity among harm reduction center providers became more pronounced during the COVID-19 pandemic.
      "*Job security and income stability are fundamental needs for all individuals. Unfortunately, these needs have received less attention in our centers. Many workers are contractors without permanent positions, leaving them vulnerable during the COVID-19 pandemic. Concerns about job loss due to closures, infection, and fear of the disease have been significant. It is crucial to address these issues with greater attention*." (Participant 4).
  8-1 Facilities
    During the COVID-19 pandemic, managers were also faced to some challenges such as “lack of adequate equipment for healthcare", and "accommodation problems".
    1-8-1 Lack of adequate equipment for healthcare
      During crises, service organizations and institutions often experience shortages of equipment and facilities due to the high number of service recipients.
      "*Due to the shortage of available beds in specialized centers during the COVID-19 pandemic, we had to quarantine patients with symptoms for 14 days before allowing them to enter the night shelter. However, the quarantine rooms lacked the necessary equipment, which posed a challenge for us as managers*." (Participant 3).
      "*During the COVID-19 pandemic, the increased demand for personal protective equipment (PPE) such as masks and gloves created challenges in the supply and demand market, making it difficult to provide these tools to the staff and clients*." (Participant 7).
    2-8-1 Accommodation problems
      During the COVID-19 pandemic, managers faced the challenge of not having a designated location for quarantining suspected COVID-19 cases.
      "*At first, we directed suspected COVID-19 cases to specialized centers. Later, we had to quarantine them in centers that were unprepared for such situations*." (Participant 6).
  9-1 Socio-cultural challenges.
    Today's society faces cultural and social issues that result in personal harm. Managers believe that these challenges have intensified during the COVID-19 pandemic. The problems include "public panic," "negative perceptions of the center and of patients," and "failure to respect human dignity."
    1-9-1 Public panic
      Due to the high number of deaths and infections, the COVID-19 pandemic was created a huge fear worldwide. This has posed a significant challenge for center managers.
      "*Our workers were apprehensive about being in harm reduction centers due to crowded environments and direct contact with vulnerable individuals*." (Participant 4).
    2-9-1 Negative perceptions of the center and of patients
      Managers have reported an increased concern from the community regarding harm reduction centers, their services, and clients during the COVID-19 pandemic.
      "*People in our city hold negative views about our service booths, often associating visitors with drug users or sex work. The negative perception has been further exacerbated by COVID-19*." (Participant 1).
      *"We faced challenges renting locations for our harm reduction centers due to neighborhood residents' objections. They blamed the centers and clients for spreading COVID-19. Addressing this negative perception was time-consuming and expensive for us."* (Participant 3).
    3-9-1 Failure to respect human dignity
      Some managers believe that clients of harm reduction centers are not adequately regarded in terms of their human dignity.
      "*During the COVID-19 pandemic, there were instances where the dignity of patients was overlooked in favor of preventing the spread of the disease from patients to the commu*nity." (Participant 2).
2. Capabilities
  During crises, individuals can demonstrate exceptional problem-solving skills. Managers have reported the emergence of their capabilities during the COVID-19 pandemic, categorized as "supervision," "executive capabilities," and "tact and diplomacy."
  1-2 Supervision
    Managers hold a crucial responsibility of overseeing the implementation process of programs and work procedures in the centers they supervise. This responsibility has become even more important during the COVID-19 pandemic. The managers' statements highlighted three subcategories: " COVID-19 registry," "staff vaccinations," and "clients screening."
    1-2-1 COVID-19 registry
      Careful monitoring and registration of patients in harm reduction centers have been one of the positive aspects of these centers.
      "*Our COVID-19 monitoring system promptly notifies us of unregistered individuals visiting our centers."* (Participant 1).
    2-2-1 Staff vaccinations
      Globally, during the COVID-19 pandemic, public vaccination mandates have been implemented in stages as a regulatory measure. This institutional endeavor has received widespread acclaim. In a recent study, managers confirmed its effectiveness.
      "*Over half of our clients and all of our staff have received at least two doses of the vaccine. It's a remarkable achievement*." (Participant 1).
    3-2-1 Clients screening
      COVID-19 screening has been mandatory in service centers such as women's harm reduction centers. Although it required significant resources, it was implemented to an acceptable level and acknowledged by the study's managers.
      "*Centers referred suspected COVID-19 cases to hospitals without any expulsions. Mobile vans were used for identification purposes in the city*." (Participant 8).
  2-2 Executive capabilities
    Harm reduction centers demonstrated excellent executive skills during the COVID-19 pandemic. Their efforts included " mobile service teams," " PPE manufacturing," and " urgent patient referral service."
    1-2-2 Mobile service teams
      Organizations resorted to unconventional methods, such as mobile teams, to provide services during the COVID-19 pandemic, as mentioned in this study.
      "*During the pandemic, mobile teams delivered condoms and face masks to customers' homes and gathering places*." (Participant 2).
    2-2-2 PPE manufacturing
      Managers highlighted the collective effort of individuals and organizations in producing protective items like masks, gloves, and coveralls during the COVID-19 pandemic.
      "*During COVID-19, some centers manufactured masks and protective gear to prevent the spread of the virus. This was commendable*!" (Participant 2).
    3-2-2 Urgent patient referral service
      Women's harm reduction centers played a crucial role in preventing the spread of COVID-19 by promptly identifying and referring patients to quarantine facilities or specialized centers. Their seamless execution of this task prevented any deaths in the centers during the pandemic and was recognized as a testament to their executive capacity.
      "*We informed all the centers under our supervision that if they come across a patient suspected to have COVID-19, they should immediately refer them to specialized centers. If the centers do not cooperate, they should report to us so that we can resolve the issue through inter-organizational coordination.*" (Participant 1).
  3-2 Tact and diplomacy
    Managers mentioned their communication capabilities during the COVID-19 pandemic, specifically in attracting crowdfunding, and attracting international aids.
    1-3-2 Attracting crowdfunding
      Managers of women's harm reduction centers collaborated with donors and established effective communication to support women in need during COVID-19.
      "*The managers of our centers secured livelihood and COVID-19 prevention health packages through effective public relations with donors*." (Participant 9).
    2-3-2 Attracting international aids
      During the COVID-19 pandemic, managers considered international participation as one of Iran's diplomatic successes.
      "*We received aid from international organizations like the UN and WHO for COVID-19 management, including diagnostic kits, medicine, vaccines, and protective gear.*" (Participant 1).
      "*Many of the COVID-19 prevention protocols we received were based on the experiences and protocols of WHO experts*." (Participant 2).

## Discussion

This study examines managers' perspectives on providing services in women's harm reduction centers during the COVID-19 pandemic in the provinces of Tehran, Khuzestan, and Kermanshah. The primary issue identified in the current research is the challenges caused by the negative consequences of the COVID-19 pandemic in managing harm reduction centers. Managers mentioned various challenges in communication, management, structure, education, finance and credit, civilization, facilities, and socio-cultural aspects. These challenges were evident in the quantitative data, showing a decrease in customer visits and service provision in some provinces. However, some health centers in Khuzestan province demonstrated improved management during the pandemy, resulting in increased overall visits, more visits from HIV-positive patients, increased condom distribution, individual counseling, and more medical and psychiatric visits. The lack of impact of the COVID-19 pandemic in these centers may be attributed to their experienced managers and their superior ability to handle crises. These challenges related to the COVID-19 pandemic were not unique to Iran, as some were also mentioned in other studies. The stigma associated with sex workers as 'prostitutes' presents a barrier for sex work organizations to access government funding during the COVID-19 pandemic, as found in a study conducted in Canada. This challenge is similar to the financial, credit, and socio-cultural issues addressed in the study [24]. The challenges related to communication, education, and financial matters discussed in the present study align with those identified in the study by Yelverton et al. (2021) conducted in South Carolina. The study highlighted technological challenges, low digital literacy, and financial difficulties for reimbursement during the COVID-19 pandemic for providing telehealth services to HIV clients [25]. Koester et al. (2022), who investigated the impact of COVID-19 on HIV/AIDS-related services in California, found that some service centers faced challenges such as the lack of manpower, ignorance of new service delivery methods, vulnerability of service providers, and reluctance to cooperate. Additionally, some centers lacked the necessary facilities to provide remote services, and clients struggled to adapt to remote HIV testing protocols during the pandemic [26]. Another result of the current study showed that the COVID-19 pandemic led to a decrease in HIV diagnostic tests in Khuzestan and Kermanshah provinces, while the numbers remained the same in Tehran province, possibly due to better access to diagnostic kits in Tehran. Condom and needle deliveries were higher before the COVID-19 pandemic in Tehran and Kermanshah, but experienced a decline during the pandemic, likely due to COVID-19 health protocols. In Khuzestan province, there was no change in deliveries. These Challenges and social problems are not unique to Iran. For instance, rehabilitation facilities in Spain also encountered challenges while acclimating their patients to quarantine measures. They had to prioritize providing food and shelter for drug users amid shortages and social challenges [3].Canadian studies also reported challenges in providing care and accessing medical professionals, shelters, and food while complying with COVID-19 health protocols [5, 18]. During the COVID-19 pandemic, several public facilities experienced a reduction in HIV-related services [6, 15–17]. During the pandemic, some harm reduction centers closed while others increased their working hours. Referrals decreased, and there were fewer services for distributing needles and conducting tests for hepatitis B, C, HIV, and tuberculosis [2, 12]. Summing up, these evidence-based studies show that the COVID-19 pandemic has presented numerous challenges for harm reduction centers worldwide. It is important to acknowledge these challenges and take steps to address them to prevent similar problems in future crises.

Participants discussed their capabilities and experience in areas such as supervision, execution, and tact and diplomacy. Also, the quantitative part of the study indicated increased capabilities in various fields such as individual and group counseling, midwifery, and medical and psychiatric visits, showing that the provinces have improved their services compared to before COVID-19 pandemic. Other studies have reported different abilities exhibited during the pandemic, such as the use of telehealth [8], increasing in-home drug allowances, providing services at home, and facilitating access to ancillary services [9, 10]. Various initiatives have been implemented in areas such as management, service delivery, organization, public health, finance and economics, and interdepartmental communication [12–14, 19–21, 26, 27]. The research demonstrates that despite the impact of COVID-19 on women's harm reduction centers in Iran, management strategies have helped mitigate negative effects. The centers have been able to maintain the quality and quantity of their services without interruption. This study focused on harm reduction centers in three Iranian provinces (Tehran, Khuzestan, and Kermanshah) to address the needs of women with HIV. Unfortunately, we were unable to obtain some statistics for the newly established centers in Kermanshah. Additionally, the service delivery and management time varied among the centers. Due to these factors, we were unable to conduct comparative statistical analyses before and during the COVID-19 pandemic in the quantitative part of the study. As a result, we presented descriptive statistics, which is a limitation of this study. The findings of this study can contribute to policy-making during crises like COVID-19, providing insights for the effective management of women's HIV harm reduction centers and mitigating the negative impacts.

## Conclusion

During the COVID-19 pandemic, there was a decrease in the number of people receiving services. Non-injecting drug users and sex workers had fewer referrals, and specific services such as group counseling were reduced. The pandemic negatively affected the performance of the centers, leading to increased challenges. To address these challenges, the managers utilized their supervisory, executive, and diplomatic skills. We hope that future research will focus on finding solutions to these challenges and evaluating their effectiveness, providing better insights for managers in this field. It is recommended to conduct future studies by comparing the number of referrals, and the types of specialized and non-specialized services during the COVID-19 pandemic Tehran, Khuzestan, and Kermanshah provinces.

## Data Availability

The data used in the current study are accessible upon reasonable request from the corresponding author.
