# Correction: The managers’ perspectives on service providing in women’s harm reduction centers during the COVID-19 pandemic: mixed method study

**DOI:** 10.1186/s12954-024-01065-z

**Published:** 2024-07-31

**Authors:** Azam Rahmani, Maryam Janatolmakan, Elham Rezaei, Malihe Tabarrai

**Affiliations:** 1 Nursing and Midwifery Care Research Center, School of Nursing and Midwifery, Tehran University of Medical Sciences, Tehran, Iran; 2 Social Development and Health Promotion Research Center, Health Institute, Kermanshah University of Medical Sciences, Kermanshah, Iran; 3 Nursing and Midwifery Care Center, Tehran University of Medical Sciences, Tehran, Iran; 4 Midwifery Department, School of Nursing and Midwifery, Tabriz University of Medical Sciences, Tabriz, Iran; 5 Department of Persian Medicine, School of Persian Medicine, Tehran University of Medical Sciences, Tehran, Iran

**Correction to: Harm Reduction Journal (2024) 21:133**

10.1186/s12954-024-01049-z

Following publication of the original article [1], the author’s name ‘Malihe Tabarrai’ was incorrectly written as ‘Malihe Tabaraei’.

The original article has been corrected.

## References

[1] Rahmani A, Janatolmakan M, Rezaei E, et al. The managers’ perspectives on service providing in women’s harm reduction centers during the COVID-19 pandemic: mixed method study. Harm Reduct J. 2024;21:133. 10.1186/s12954-024-01049-z. 38997734 10.1186/s12954-024-01049-z PMC11241914

